# MicroRNA-495 serves as a diagnostic biomarker in patients with sepsis and regulates sepsis-induced inflammation and cardiac dysfunction

**DOI:** 10.1186/s40001-019-0396-3

**Published:** 2019-11-26

**Authors:** Hailei Guo, Liying Tang, Jianjun Xu, Cai Lin, Xiangwei Ling, Caijiao Lu, Zhengjun Liu

**Affiliations:** 1 Department of Burns Surgery, The First Affiliated Hospital of Wenzhou Medical University, Nanbaixiang Street, Wenzhou, 325000 Zhejiang People’s Republic of China; 2 Department of Burns Surgery, Jinan Central Hospital Affiliated to Shandong University, Jinan, 250013 People’s Republic of China; 3 Department of Rehabilitation, Wenzhou Hospital of Traditional Chinese Medicine Affiliated to Zhejiang, Chinese Medical University, Wenzhou, 32500 People’s Republic of China

**Keywords:** MiR-495, Sepsis, Diagnosis, Cardiac dysfunction, Inflammatory

## Abstract

**Background:**

Sepsis leads to severe inflammatory and cardiac dysfunction. This study aimed to explore the clinical value of miR-495 in sepsis, as well as its role in sepsis-induced inflammation and cardiac dysfunction.

**Methods:**

105 sepsis patients were recruited; receiver operating characteristic (ROC) curve was plotted to assess the diagnostic value of miR-495 in sepsis. A model of sepsis in rats was created via performing cecal ligation and puncture (CLP). After modeling, the cardiac function, including left ventricular systolic pressure (LVSP), left ventricular end diastolic pressure (LVEDP) and maximum rate of rise/fall of left ventricle pressure (± dp/dt_max_), and serum cardiac troponin I (CTn-I), creative kinase isoenzyme MB (CK-MB) were detected. The blood cytokines levels including TNF-α, IL-6, IL-1β were also measured. Quantitative real-time PCR (qRT-PCR) was used for the measurement of the expression level of miR-495.

**Results:**

MiR-495 was significantly downregulated in sepsis patients, especially patients who suffered from septic shock (SS). MiR-495 expression was negatively associated with Scr, WBC, CRP, PCT, APACHE II score and SOFA score. MiR-495 could distinguish patients with SS from non-SS patients. MiR-495 and SOFA score were better indictors for the occurrence of cardiac dysfunction in sepsis patients. In CLP-induced sepsis model. CLP rats experienced deterioration of LVSP, LVEDP, ± dp/dt_max_, and had a rise in serum CTn-I, CK-MB, TNF-α, IL-6 and IL-1β, which were improved by miR-495 agomir injection.

**Conclusions:**

MiR-495 might be a potential diagnostic biomarker for sepsis patients, and overexpression of miR-495 alleviated sepsis-induced inflammation and cardiac dysfunction.

## Background

Sepsis is defined as the imbalance of the host response to infection, which can lead to life-threatening organ dysfunction [1]. Sepsis can lead to a severe systemic inflammatory response, even shock [2]. It has been reported that cardiac dysfunction is a major complication of patients with sepsis, and is closely associated with sepsis-induced mortality [3]. Although major improvements have been made in critical care medicine in recent decades, the incidence and mortality of sepsis are still high [4, 5]. Current studies focus on the identification of early biomarkers to predict sepsis outcome, including serum C-reactive protein (CRP), procalcitonin (PCT) levels [6], and acute physiology and chronic health evaluation II scores (APACHE II) and sequential organ failure assessment scores (SOFA) [7]. Novel biomarkers are beneficial for the early diagnosis and accurate assessment of sepsis. Furthermore, it may be helpful for fully understanding the mechanisms underlying sepsis.

MicroRNAs (miRNAs) are a kind of endogenous noncoding small RNAs with a length of approximately 22 nucleotides [8]. Currently, a large number of miRNAs have been identified to be potential novel biomarkers for early diagnosis or prognosis of various human diseases [9]. The use of miRNAs has also been widely reported to diagnose or stage sepsis in the critically ill [10–12]. Many reports demonstrated that miR-495 functions as a tumor suppressor, and modulates proliferation and migration of several tumor cells, such as colon cancer, gastric cancer, bladder cancer and so on [13–15]. Recent studies have already suggested the involvement of miR-495 in the inflammatory response of several human diseases, such as ankylosing spondylitis (AS), inflammatory bowel disease [16, 17]. Notably, Wang et al. [18] reported that miR-495 is involved in the regulation of cardiac fibrosis, which plays a crucial role in sepsis-induced cardiac dysfunction. But the role of miR-495 in sepsis has not been reported.

Therefore, this study explored the expression and clinical role of miR-495 in sepsis, and further investigated its role in sepsis-induced inflammation and cardiac dysfunction.

## Materials and methods

### Study population and sample collection

This study was approved by the Committee on Ethics of the First Affiliated Hospital of Wenzhou Medical University. Written informed consent was obtained from all participants or their families. Following the Surviving Sepsis Campaign: International Guidelines for Management of Severe Sepsis and Septic Shock, 2012 [19], a total of 105 sepsis patients were recruited, who were admitted to intensive care units (ICUs) of the First Affiliated Hospital of Wenzhou Medical University from May 2016 to August 2017. Patients were excluded for any of the following reasons: less than 18 years old; in an immunocompromised state; pregnant; lactating; human immunodeficiency virus (HIV)-positive; in end-of-life conditions. All sepsis patients were further classified into septic shock (SS) group and non-septic shock (non-SS) group. SS patients were defined as sepsis patients who have persistent hypotension that requires vasopressors to maintain a MAP ≥ 65 mmHg and who have a serum lactate level > 2 mmol/L despite adequate volume resuscitation [20]. Among these sepsis patients, 71 were included as non-SS group, and other 34 patients were included in SS group. During treatment, the heart function of all patients was monitored and recorded. Additionally, 100 healthy age and gender-matched healthy individuals were collected as control group, who had no history of severe infection or malignancies, or other obvious abnormalities by physical examination. Within 24 h of admission to intensive care unit (ICU), the APACHE II score and SOFA score of sepsis patients were assessed, and blood samples were collected. The clinical data were recorded from each participant, including age, gender, body mass index (BMI), serum creatinine (Scr), albumin, white blood cell (WBC), CRP, PCT.

### Animal models of sepsis

Male Sprague–Dawley rats weighing 250–300 g were purchased from the Laboratory Animal Center of Nanjing Medical University (Nanjing, Jiangsu Province, China), and housed under appropriate conditions (25 ± 2 °C and 12 h light/dark cycle) with free access to water and food before the experiment. All of the animal procedures were performed in accordance with the Guide for the Care and Use of Laboratory Animals and approved by the Medical Ethics Committee of the First Affiliated Hospital of Wenzhou Medical University. Cecal ligation and puncture (CLP) were performed to induce sepsis as described previously [21]. In brief, the rats were anesthetized with sodium pentobarbital (50 mg/kg, Sigma, St. Louis, MO, USA). A midline incision (about 2 cm) was made on the anterior abdomen, then the cecum was exposed and the ligation was at the position of 30% of the cecum. Before closing the abdominal cavity, the cecum was punctured twice with a sterile 18-gauge needle to extrude the fecal material. The sham control rats underwent the same procedure without ligation and puncture. After surgery, 1-mL normal saline was injected to each rat for resuscitation.

### Animal grouping

The rats were divided into four groups as follows: sham group (sham control rats, 1-mL normal saline was injected intravenously 24 h before the surgery); CLP group (CLP-treated rats, 1-mL normal saline was injected intravenously 24 h before the surgery); miR-495 negative control (NC) group (CLP-treated rats, 10 μg miR-495 NC sequence was injected intravenously 24 h before the surgery); miR-495 agomir group (CLP-treated rats, 10 μg miR-495 agomir was injected intravenously 24 h before the surgery). MiR-495 NC and miR-495 agomir were synthesized and provided by GenePharma, Shanghai, China. After molding and injection, each group had at least 8 viable individuals.

### Cardiac function and blood cytokines assessments

After surgery, the cardiac function of rats was detected. The LVSP, LVEDP and maximum rate of rise/fall of left ventricle pressure (± dp/dt_max_) were measured using the MFLab 3.01 package in FDP-1 HRV & BRS analysis system (Shanghai Jialong, Shanghai, China). 5-mL venous blood sample was collected from each rat. Enzyme-linked immunosorbent assay (ELISA) was performed to measure the content of serum cardiac troponin I (CTn-I) and creative kinase isoenzyme MB (CK-MB), and serum levels of tumor necrosis factor alpha (TNF-α), interleukin 6 (IL-6), IL-1β.

### RNA extraction and quantitative real-time polymerase chain reaction (qRT-PCR)

After all the tests were finished, the rats were killed and the tissues from myocardium were collected. Total RNA was isolated using Trizol reagent (Life Technologies Corporation, Carlsbad, CA, USA). TaqMan miRNA reverse transcription kit (Applied Biosystems, Foster City, CA, USA) was used for reverse transcription. The Real-Time PCR analysis was performed using the One Step SYBR^®^ PrimeScript^®^PLUS RT-RNA PCR Kit (TaKaRa Biotechnology, Dalian, China). The relative expression of miR-495 was determined in relation to U6 by the comparative delta CT (2^−ΔΔCt^) method.

### Statistical analysis

All data analyses were conducted using SPSS version 18.0 software (SPSS Inc., Chicago, IL) and GraphPad Prism 5.0 software (GraphPad Software, Inc., USA). The data were expressed as mean ± standard deviation (SD). Differences between groups were compared using the Chi-square test, student’s *t* test or one-way ANOVA analysis. Correlations were determined by Spearman’s rank correlation test. Binary logistic regression analysis was used to evaluate the association of different variables with the occurrence of cardiac dysfunction in sepsis patients. Receiver operating characteristic (ROC) curve was plotted, and the area under curve (AUC) with 95% confidence interval (CI) was calculated to assess the discrimination ability of miR-495 expression between sepsis patients and healthy controls. *P* < 0.05 was considered to indicate a statistically significant difference.

## Results

### Clinical characteristics of study objects

There were 100 healthy controls and 105 sepsis patients with the mean age of 56.39 ± 10.20 and 55.06 ± 9.17, respectively (Table 1). No significant difference was detected for age, gender and BMI between the two groups (all *P* > 0.05). The serum levels of Scr, WBC, CRP and PCT were higher in sepsis patients than that in healthy controls, whereas the serum albumin level was lower in patients group, and all differences reached significant levels (all *P* < 0.001). And the mean value of APACHE II score and SOFA score in sepsis patients were 12.92 ± 3.22 and 5.28 ± 1.31, respectively.

**Table 1:** Comparison of the baseline data between the two groups of study objects

Parameters	Health (n = 100)	Sepsis (n = 105)	*P* value
Age (year)	56.39 ± 10.20	55.06 ± 9.17	0.327
Gender (male/female)	61/39	59/46	0.485
BMI (kg/m^2^)	20.54 ± 1.87	20.52 ± 1.97	0.955
Scr (mg/dL)	1.10 ± 0.32	1.69 ± 0.43	< 0.001
Albumin (g/L)	40.74 ± 4.37	25.69 ± 4.34	< 0.001
WBC (× 10^9^/L)	7.53 ± 1.61	18.73 ± 6.29	< 0.001
CRP (mg/L)	6.03 ± 2.46	94.90 ± 26.23	< 0.001
PCT (ng/mL)	0.05 ± 0.02	12.88 ± 5.24	< 0.001
APACHE II score	–	12.92 ± 3.22	–
SOFA score	–	5.28 ± 1.31	–

*BMI* body mass index, *Scr* serum creatinine, *WBC* white blood cell, *CRP* C-reactive protein, *PCT* procalcitonin, *APACHE* acute physiology and chronic health evaluation, *SOFA* sequential organ failure assessment

### Serum level of miR-495 in sepsis patients

**Fig. 1:**
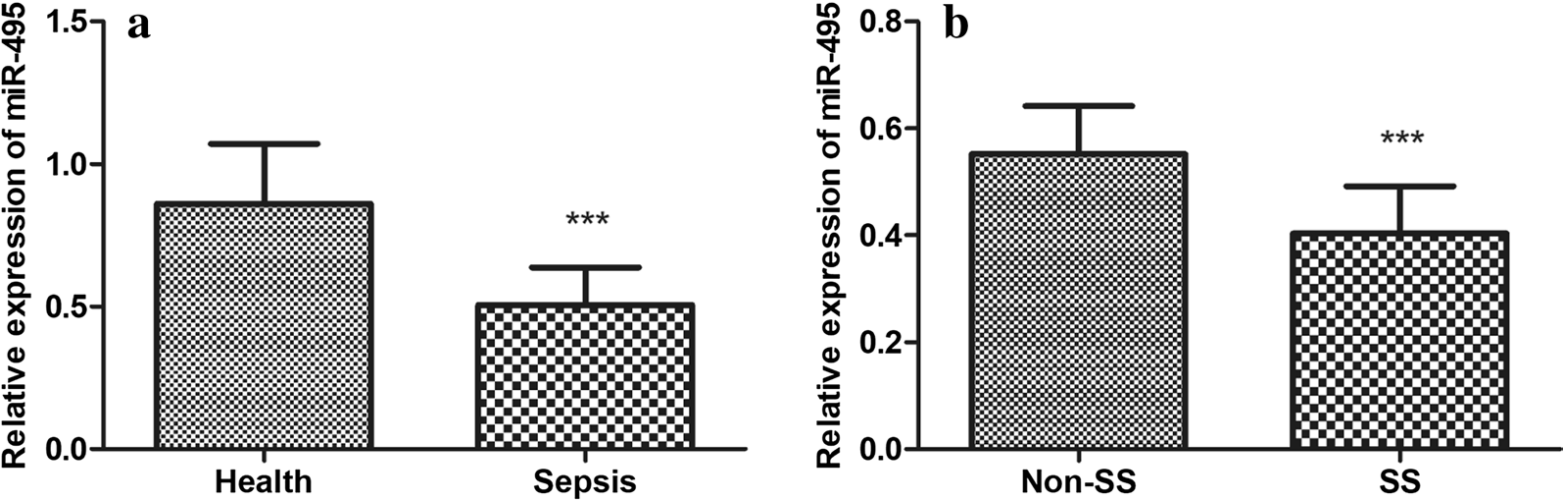
The serum expression level of miR-495 in sepsis patients and healthy controls were measured using qRT-PCR. **a** MiR-495 was significantly downregulated in patients group compared with healthy controls. **b** MiR-495 level in SS group was significantly lower than that in non-SS group. ***P < 0.001

The serum expression level of miR-495 in sepsis patients and healthy controls were measured using qRT-PCR. As shown in Fig. 1a, serum miR-495 was significantly downregulated in patients group compared with healthy controls (*P* < 0.001). Additionally, we further compared the miR-495 expression levels between non-SS and SS patients. It was found that miR-495 level in SS group was significantly lower than that in non-SS group (Fig. 1b, *P* < 0.001).

### Correlation of miR-495 expression with clinicopathological features of sepsis patients

Regarding all the clinicopathological features in sepsis patients, miR-495 expression was negatively associated with Scr, WBC, CRP, PCT, APACHE II score and SOFA score (all *P* < 0.05, Table 2). However, miR-495 expression was not associated with age, gender, BMI and albumin (all *P* > 0.05). These results indicated that serum miR-495 expression level was negatively associated with severity in sepsis patients.

**Table 2:** Correlation of miR-495 relative expression with clinical characteristics

Parameters	MiR-495 expression	
	*P* value	Correlation coefficient (*r*)
Age	0.902	0.012
Gender	0.519	− 0.064
BMI	0.435	0.077
Scr	0.035	− 0.206
Albumin	0.192	0.128
WBC	< 0.001	− 0.573
CRP	< 0.001	− 0.509
PCT	< 0.001	− 0.404
APACHE II score	< 0.001	− 0.500
SOFA score	< 0.001	− 0.523

*BMI* body mass index, *Scr* serum creatinine, *WBC* white blood cell, *CRP* C-reactive protein, *PCT* procalcitonin, *APACHE* acute physiology and chronic health evaluation, *SOFA* sequential organ failure assessment

### Diagnostic value of miR-495 expression for patients with sepsis

**Fig. 2:**
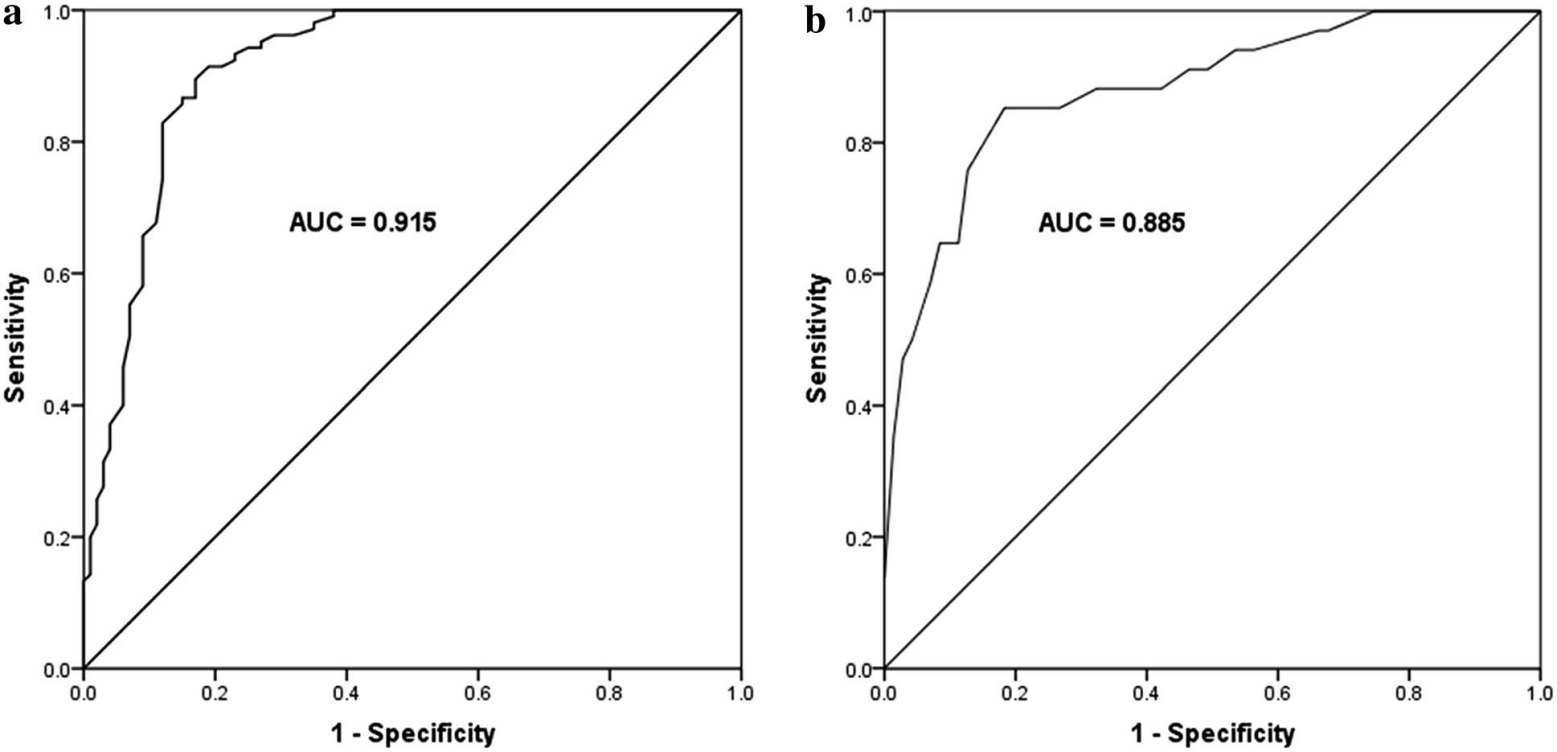
Receiver operating characteristic (ROC) curve for patients with sepsis and healthy controls. **a** The diagnostic value of serum miR-495 level in sepsis patients compared with healthy controls. The area under curve (AUC) value was 0.915 yield the sensitivity of 89.5% and specificity of 83.0% at the cutoff value of 0.655. **b** The diagnostic value of miR-495 level in SS patients compared with non-SS patients. The AUC value was 0.885 yield the sensitivity of 85.3% and specificity of 87.3% at the cutoff value of 0.475

The ROC curve was conducted to assess the diagnostic value of miR-495 in sepsis with the serum miR-495 expression in sepsis patients, and corresponding healthy individuals were used as control. As shown in Fig. 2a, the diagnostic value of serum miR-495 level in sepsis patients compared with healthy controls was assessed. It was found that the AUC value was 0.915 yielding the sensitivity of 89.5% and specificity of 83.0% at the cutoff value of 0.655. These results indicated that serum miR-495 levels could be used to distinguish sepsis patients from healthy individuals. Additionally, the diagnostic value of miR-495 level in SS patients compared with non-SS patients was further evaluated. As shown in Fig. 2b, the AUC value was 0.885 yielding the sensitivity of 85.3% and specificity of 87.3% at the cutoff value of 0.475. It indicated that miR-495 could distinguish patients with SS from non-SS patients.

### Association of miR-495 expression with the occurrence of cardiac dysfunction in sepsis patients

According to the results of heart function monitor, the sepsis patients were divided into two groups: the normal group (n = 39) and the cardiac dysfunction group (n = 66). Then, the logistic regression analysis was performed to evaluate the association miR-495 expression with the occurrence of cardiac dysfunction. As shown in Table 3, it was observed that downregulation of miR-495 (OR = 0.237, 95% CI 0.082–0.689, *P* = 0.08) and high level of SOFA score (OR = 3.291, 95% CI 1.267–8.552, *P* = 0.014) were independent factors for the occurrence of cardiac dysfunction in sepsis patients. It was concluded that miR-495 and SOFA scores were better indictors for the occurrence of cardiac dysfunction in sepsis patients.

**Table 3:** Association of different variables with the occurrence of heart dysfunction in sepsis patients

Variables	OR	95% CI	*P* value
MiR-495	0.237	0.082–0.689	0.008
Age	1.050	0.440–2.505	0.912
Gender	1.198	0.494–2.906	0.689
BMI	1.244	0.442–3.505	0.679
Scr	1.090	0.453–2.623	0.848
Albumin	0.726	0.282–1.869	0.507
WBC	0.749	0.249–2.259	0.608
CRP	1.289	0.472–3.520	0.621
PCT	2.199	0.717–6.751	0.168
APACHE II score	1.978	0.757–5.163	0.164
SOFA score	3.291	1.267–8.552	0.014

*BMI* body mass index, *Scr* serum creatinine, *WBC* white blood cell, *CRP* C-reactive protein, *PCT* procalcitonin, *APACHE* acute physiology and chronic health evaluation, *SOFA* sequential organ failure assessment

### MiR-495 regulates sepsis-induced cardiac dysfunction in rat models

**Fig. 3:**
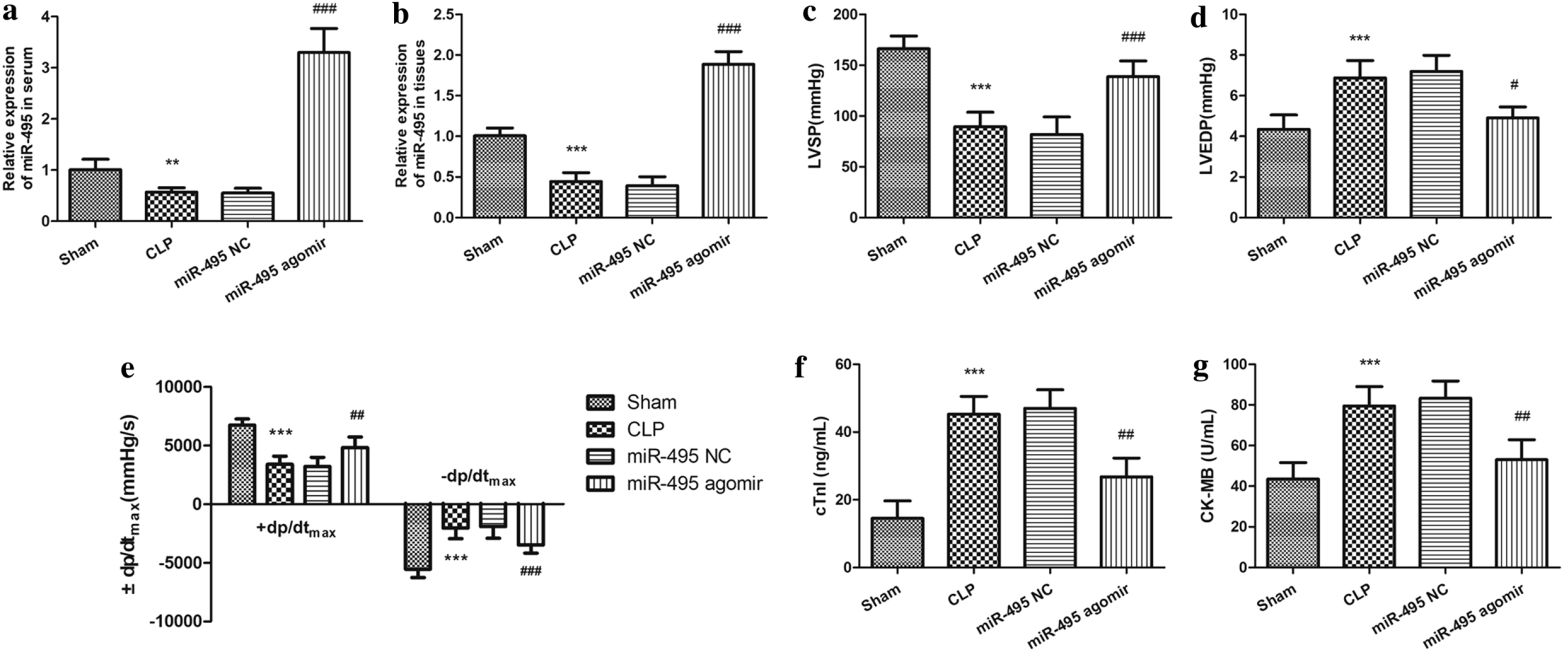
The effect of miR-495 on sepsis-induced cardiac dysfunction in sepsis modeling rats. **a**–**b**. MiR-495 expression level in rat serum and myocardium after CLP modeling. **c**–**e**. Comparison of cardiac hemodynamic after sepsis modeling in rats. **f**–**g**. Comparison of serum myocardial enzyme indexes in sepsis modeling rats. **P < 0.01, ***P < 0.001, compared with sham group;^#^*P* < 0.05, ^##^*P* < 0.01, ^###^*P* < 0.001, compared with CLP group

To investigate the potential role of miR-495 in sepsis-induced cardiac dysfunction, sepsis model was constructed in rats. As shown in Fig. 3a, b, miR-495 expression was significantly downregulated in both rat serum and myocardium after CLP modeling, which was reversed by miR-495 agomir injection (all *P* < 0.01). It was suggested that miR-495 agomir injection could effectively upregulate miR-495 expression in sepsis rats. Furthermore, the cardiac function of rats was assayed. It was found that LVSP and + dp/dt_max_ significantly decreased, whereas LVEDP and − dp/dt_max_ remarkably increased in the CLP group compared with the sham group (all *P* < 0.01, Fig. 3c–e). Additionally, serum cTnI, CK-MB also showed remarkably increasing trend in the CLP group compared with the sham group (all *P* < 0.01, Fig. 3f, g). These results suggested that myocardial dysfunction occurred in the rat sepsis model. However, it was noted that overexpression of miR-495 significantly reversed the effect of CLP surgery on myocardial function of rats, which was indicated by the increase of LVSP and + dp/dt_max_ and the decrease of LVEDP and − dp/dt_max_, as well as serum cTnI, CK-MB levels. We concluded that miR-495 may be involved in the regulation of myocardial function of sepsis.

### MiR-495 regulates sepsis-induced inflammation in rat models

**Fig. 4:**
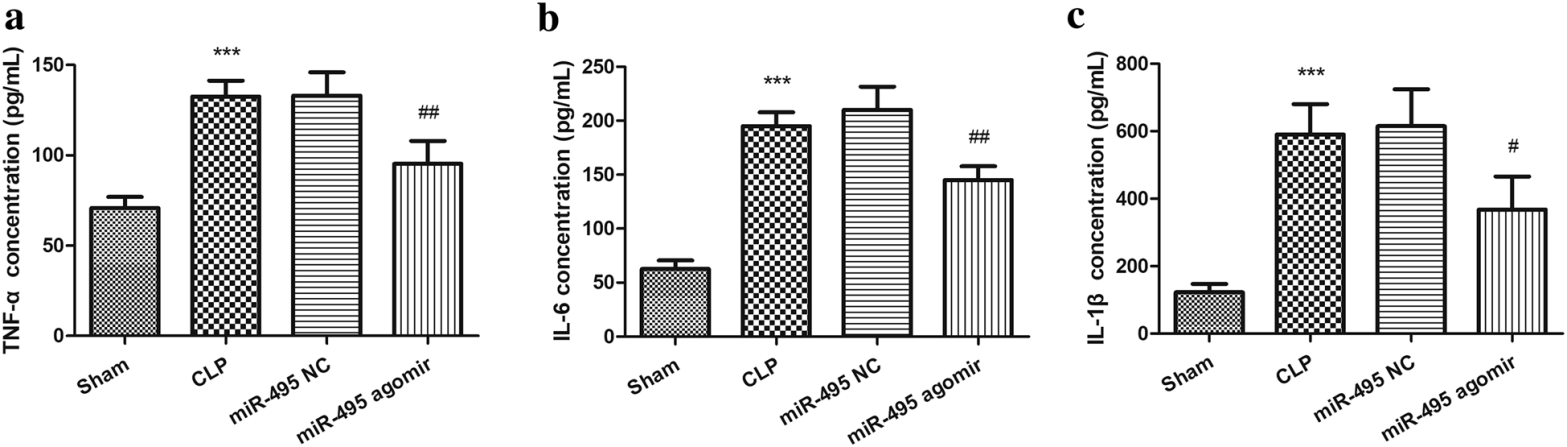
Serum indexes of inflammatory response after sepsis modeling in rats. ***P < 0.001, compared with sham group; ^#^*P* < 0.05, ^##^*P* < 0.01, compared with CLP group

To explore the effect of miR-495 on sepsis-induced inflammation, the levels of three inflammatory factors in rat serum were detected. It was observed that serum TNF-α, IL-6 and IL-1β levels were all increased significantly in CLP group compared with the sham group, which were effectively attenuated by miR-495 agomir injection (all *P* < 0.05, Fig. 4). These results indicated that CLP-induced sepsis presented obvious inflammatory reaction, but miR-495 overexpression could relive sepsis-induced inflammation.

## Discussion

Sepsis is a relatively common SIRS in the intensive care unit (ICU), and has a high rate of fatality. The immediate diagnosis and intervention are beneficial for the satisfactory clinical outcomes of sepsis patients. A growing number of researchers are applying themselves to identify the biomarkers for the early diagnosis of sepsis. Currently, PCT is approved to be used as a biomarker to differentiate culture-negative and culture-positive sepsis from non-infectious SIRS [22]. Additionally, several other diagnostic and prognostic biomarkers have been identified in sepsis, such as CRP, serum lactate, and IL-6, but the sensitivity or specificity is not high enough for enhancing the timely intervention of sepsis [23–25].

Dysregulated miRNA expression has been widely reported to be associated with the clinical features of SIRS, critically ill polytrauma, and sepsis [26, 27]. In the present study, miR-495 was determined to be downregulated in sepsis patients for the first time. Previous study has suggested that miR-495 is an important miRNA involved in multiple kinds of cancers, and is also associated with the proliferation, invasion, metastasis and drug resistance of tumor cells. For example, in endometrial cancer (EC), miR-495 was downregulated in tumor tissues, and overexpression of miR-495 markedly inhibited tumor cell proliferation and promoted cell apoptosis via targeting PIK3R1 [28]. In oral squamous cell carcinoma (OSCC), miR-495 was also reported to play an antitumor effect through regulating OSCC cell growth and metastasis [29]. In healthy cells, miR-495 was also reported to participate in a variety of developmental, apoptotic, immune and inflammatory mechanisms [30]. Additionally, miR-495 has also been identified to be a diagnostic or prognostic biomarker in several diseases, and might be a potential therapeutic target for the treatment of diseases [31–33]. Our study suggested that serum miR-495 expression level showed close correlation with several inflammatory marker in sepsis patients, and ROC curve analysis suggested serum miR-495 to be a potential diagnostic biomarker to distinguish sepsis patients from healthy individuals. Furthermore, miR-495 was also overexpressed in SS patients, and miR-495 could distinguish patients with SS from non-SS patients. We concluded that miR-495 might be associated with the severity of sepsis.

Previous study has demonstrated that cardiac dysfunction is a major complication in sepsis patients, and is closely related to sepsis-induced mortality [34]. Approximately, half of the patients with severe sepsis suffer myocardial depression to some extent, with the high mortality of 70–90% [35]. MiR-495 has been widely reported to play a crucial role in various cardiac diseases, and involved in the regulation of myocardial function [18, 36]. Notably, miR-495 is involved in the regulation of cardiac fibrosis, which plays a crucial role in sepsis-induced cardiac dysfunction [18, 37]. In the present study, logistic regression analysis was performed to evaluate the association miR-495 expression with the occurrence of cardiac dysfunction. It was observed that downregulation of miR-495 and high level of SOFA score were independent factors for the occurrence of cardiac dysfunction in sepsis patients. Furthermore, we established a sepsis model in rats to explore the role of miR-495 in sepsis-induced cardiac dysfunction. Our study showed that the sepsis model in rats developed severe cardiac dysfunction, but it was significantly improved by miR-495 upregulating, which was consistent with the previous evidences. We concluded that miR-495 may be involved in the regulation of myocardial function of sepsis.

MiR-495 is an inflammatory-related miRNA and has been reported to play an anti-inflammatory role in several human diseases. Du et al. [16] reported that the level of miR-495 was decreased in AS patients, and the cell experiment suggested that miR-495 suppressed the inflammatory response of AS by downregulating TNF-α, IL-1β and IL-6. Another study about cardiac microvascular endothelial cell (CMEC) injury reported that miR-495 ameliorated CMEC injury and inhibited inflammatory reaction via suppressing the NLRP3 inflammasome signaling pathway, and NLRP3 was proved to be the target gene of miR-495 [36]. Consistent with the previous evidence, the present study results demonstrated that the CLP rats had a significant rise in serum TNF-α, IL-6 and IL-1β, which were dropped by miR-495 overexpression. It is well known that these inflammatory factors are major contributors to the development of cardiac dysfunction in sepsis [38, 39]. These results accompany with the previous evidence, we concluded that miR-495 plays a significant role against the inflammatory response, and the anti-inflammatory effect of miR-495 might be associated with its cardioprotective effects on sepsis-induced cardiac dysfunction. However, there were some limitations in our study. The study population was relatively small, and a much larger group is needed to verify the present results. Additionally, further studies are warranted to investigate the concrete mechanism underlying the role of miR-495 in heart function of sepsis.

In summary, our experiments demonstrated that miR-495 was downregulated in sepsis patients, and was a better indicator for the occurrence of cardiac dysfunction in sepsis patients. Overexpression of miR-495 alleviated sepsis-induced inflammation and cardiac dysfunction. MiR-495 might be a potential diagnostic biomarker and will facilitate early treatment of sepsis.

## Data Availability

All data generated during this study are included in this published article.

## Abbreviations

CLP: cecal ligation and puncture
LVSP: left ventricular systolic pressure
LVEDP: left ventricular end diastolic pressure
CTn-I: cardiac troponin I
qRT-PCR: quantitative real-time PCR
CK-MB: creative kinase isoenzyme MB
SIRS: systemic inflammatory response syndrome
CRP: C-reactive protein
PCT: procalcitonin
APACHE II: acute physiology and chronic health evaluation II scores
SOFA: sequential organ failure assessment scores
miRNAs: MicroRNAs
AS: ankylosing spondylitis
ICUs: intensive care units
HIV: human immunodeficiency virus
BMI: body mass index
Scr: serum creatinine
WBC: albumin, white blood cell
CLP: cecal ligation and puncture
NC: negative control
ELISA: enzyme-linked immunosorbent assay
TNF-α: tumor necrosis factor alpha
IL-6: interleukin 6
qRT-PCR: quantitative real-time polymerase chain reaction
SD: standard deviation
ROC: receiver operating characteristic
AUC: the area under curve
CI: confidence interval
CMEC: cardiac microvascular endothelial cell
